# Refinement of response assessment in neuro-oncology (RANO) using non-enhancing lesion type and contrast enhancement evolution pattern in IDH wild-type glioblastomas

**DOI:** 10.1186/s12885-021-08414-2

**Published:** 2021-06-01

**Authors:** Hye Hyeon Moon, Ho Sung Kim, Ji Eun Park, Young-Hoon Kim, Jeong Hoon Kim

**Affiliations:** 1 Department of Radiology and Research Institute of Radiology, University of Ulsan College of Medicine, Asan Medical Center, 43 Olympic-ro 88, 86 Asanbyeongwon-Gil, Songpa-Gu, Seoul, 05505 Republic of Korea; 2 Department of Neurosurgery, University of Ulsan College of Medicine, Asan Medical Center, 86 Asanbyeongwon-Gil, Songpa-Gu, Seoul, 05505 Republic of Korea

**Keywords:** Progression, Glioblastoma, MRI, Non-enhancing lesion, Contrast enhancement

## Abstract

**Background:**

Updated response assessment in neuro-oncology (RANO) does not consider peritumoral non-enhancing lesion (NEL) and baseline (residual) contrast enhancement (CE) volume. The objective of this study is to explore helpful imaging characteristics to refine RANO for assessing early treatment response (pseudoprogression and time-to-progression [TTP]) in patients with IDH wild-type glioblastoma.

**Methods:**

This retrospective study enrolled 86 patients with IDH wild-type glioblastoma who underwent consecutive MRI examinations before and after concurrent chemoradiotherapy (CCRT). NEL was classified as edema- or tumor-dominant type on pre-CCRT MRI. CE evolution was categorized into 4 patterns based on post-operative residual CE (measurable vs. non-measurable) and CE volume change (same criteria with RANO) during CCRT. Multivariable logistic regression, including clinical parameters, NEL type, and CE evolution pattern, was used to analyze pseudoprogression rate. TTP and OS according to NEL type and CE evolution pattern was analyzed by the Kaplan–Meier method.

**Results:**

Pseudoprogression rate was significantly lower (chi-square test, *P* = .047) and TTP was significantly shorter (hazard ratio [HR] = 2.03, *P* = .005) for tumor-dominant type than edema-dominant type of NEL. NEL type was the only predictive marker of pseudoprogression on multivariate analysis (odds ratio = 0.26, *P* = .046). Among CE evolution patterns, TTP and OS was shortest in patients with residual CE compared with those exhibiting new CE (HR = 4.33, *P* < 0.001 and HR = 3.71, *P* = .009, respectively). In edema-dominant NEL type, both TTP and OS was stratified by CE evolution pattern (log-rank, *P* = .001), whereas it was not in tumor-dominant NEL.

**Conclusions:**

NEL type improves prediction of pseudoprogression and, together with CE evolution pattern, further stratifies TTP and OS in patients with IDH wild-type glioblastoma and may become a helpful biomarker for refining RANO.

**Supplementary Information:**

The online version contains supplementary material available at 10.1186/s12885-021-08414-2.

## Background

The Response Assessment in Neuro-oncology (RANO) criteria are accurate and reproducible in the radiologic assessment of treatment response in patients with glioblastoma [1]. RANO criteria focus primarily on objective measurements of contrast enhancement (CE), whereas the importance of non-contrast enhancing components of tumors is frequently overlooked [2–4]. Peritumoral non-enhancing lesions (NELs), which appear as hyperintense lesions on T2-weighted fluid-attenuated inversion recovery (FLAIR) imaging [5], usually consist of mixtures of edema and tumor [6]. Because glioblastomas are infiltrative, NELs can affect patient prognosis [7–9], as they represent the portion of tumors extending beyond the contrast-enhanced margins.

Although advanced magnetic resonance imaging (MRI) protocols, including perfusion MRI, diffusion-weighted imaging (DWI), and MR spectroscopy, have shown potential for distinguishing between tumor and edema in NELs [10, 11], these methods require additional postprocessing method and are not easily applied in clinical practice [12, 13]. Attempts have been made to distinguish infiltrative tumor from edema in NEL by their morphology on anatomical MRI [14]. Tumor-dominant NELs have several characteristic imaging features, including relatively mild FLAIR hyperintensity, gray matter involvement, eccentric extension not accounted for by anatomic constraints, focal parenchymal expansion, and mass effect [14–16].

The post-operative residual CE volume is another important prognostic marker, as extent of surgical resection has been associated with overall survival [17, 18]. However, RANO criteria did not substratify evolution of CE after treatment, whether responses are determined by comparisons with residual measurable CE or non-measurable CE. For example, stable disease (SD) can indicate an absence of change in residual measurable CE on follow-up examination, or no CE at all, without change on follow-up. Assessment of response to CCRT may be refined by evaluating CE and NEL before and after treatment. The present study evaluated whether categorizing NEL type and CE evolution pattern during CCRT can better predict time to progression (TTP) and pseudoprogression in patients with isocitrate dehydrogenase (IDH) wild-type glioblastoma.

## Materials and methods

### Study population

**Fig. 1:**
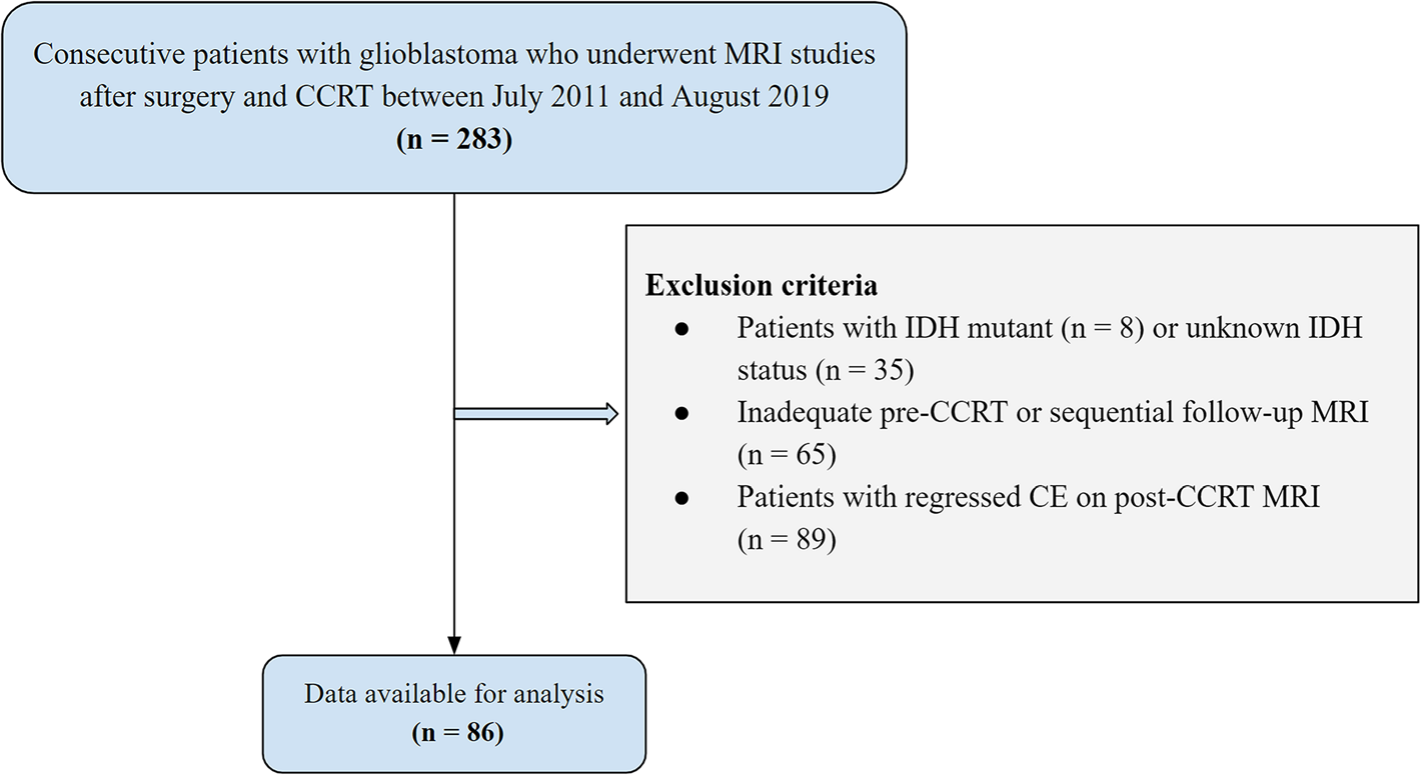
Study flow chart showing included and excluded patients. CCRT = concurrent chemoradiotherapy; IDH = isocitrate dehydrogenase

This retrospective clinical study was approved by the institutional review board (IRB) of Asan Medical Center (local approval number: 2019–0135) and was conducted in compliance with the U.S. Health Insurance Portability and Accountability Act (HIPAA) regulations and the Declaration of Helsinki. The requirement of written informed consent was waived in this retrospective study as per IRB-approval protocol. The Asan Medical Center database was retrospectively reviewed to identify consecutive patients who were confirmed as having glioblastoma between July 2011 and August 2019, and were evaluated by MRI after surgery followed by CCRT. Patients were included if they (i) had been histologically diagnosed with IDH wild-type glioblastoma according to the World Health Organization (WHO) criteria; (ii) underwent adjuvant CCRT that included six cycles of temozolomide (TMZ) treatment after surgical resection or biopsy; (iii) had been evaluated by MRI, including contrast-enhanced T1-weighted imaging (CE-T1WI) and FLAIR imaging, within 2 weeks after surgery or biopsy and before CCRT, and again 4 weeks after completing CCRT; (iv) had a newly developed or persistent CE on post-CCRT MRI; and (vi) were sequentially followed-up by contrast-enhanced MRI at 2–3 month intervals for at least 12 months to confirm the final diagnosis of pseudoprogression and progression. The protocol of this retrospective study was approved by the institutional review board of Asan Medical Center, which waived the requirement for patient informed consent (approval number: 2019–1259). A study flowchart is shown in Fig. 1.

### Molecular/genomic analysis

#### IDH mutation status analysis

Immunohistochemistry for IDH1 (R132H) protein expression was used as the reference standard for this study. In patients older than 55 years with typical newly identified glioblastoma at diagnosis, a negative immunohistochemistry result for IDH1 (R132H) expression was sufficient for IDH wild-type glioblastoma classification, according to the European Association of Neuro-Oncology guidelines. In patients younger than 55 years at diagnosis, DNA pyrosequencing for mutations in IDH1 or IDH2 genes was performed [19]*.*

#### MGMT promoter methylation analysis

MGMT promoter methylation status was evaluated using a methylation-specific polymerase chain reaction (PCR) assay [20]. The genomic DNA was extracted from an unstained tissue slide made from paraffin-embedded blocks of tumor tissue. DNA methylation status of CpG (cytosine and guanine nucleotides separated by one phosphate nucleotide) islands at the MGMT promoter was evaluated using the methylation-specific PCR assay with some modifications [21]. Bisulfite-treated unmethylated DNA and methylated DNA (Qiagen, Hilden, Germany) were used as low and high controls. PCR products were separated on 8% polyacrylamide gels and stained with ethidium bromide. Subsequently, they were examined under ultraviolet illumination. The clinical information was blinded with a computerized barcode during all these processes.

### Reference standard for final diagnosis

Pseudoprogression and true progression were pathologically confirmed on second-look operations when clinically indicated. In the absence of second-look operations, consecutive clinicoradiological diagnoses were made by consensus between two neuro-oncologists (J.H.K., Y.H.K; with 28 and 10 years of clinical experience in neuro-oncology, respectively) according to RANO criteria [4]. Pseudoprogression was defined as an increase in CE or any new lesion followed by stabilization or regression without any changes in treatment for at least 6 months after surgery and completion of CCRT [4]. True progression was defined as the occurrence of any new lesion outside the radiation field or a gradual increase in CE size on more than two subsequent follow-up MRI examinations performed at 2–3 month intervals and requiring a prompt change in treatment [4]. TTP was defined as the time from the date of initial diagnosis to the date of first documented progression [22]. The co-primary endpoint was overall survival (OS). OS was calculated from the day of histopathologic diagnosis until the day of death as obtained from the national health care data linked to our hospital. Patients who were alive at the time of analysis (*n* = 17, 19.7%) with right-censored data were included in the analysis. All patients were followed up every 3–6 months.

### Magnetic resonance imaging

All MRI evaluations were performed using a 3-T unit (Ingenia 3.0 CX; Philips Healthcare, Best, the Netherlands) with a 16-channel head coil, and included the following sequences: T2-weighted, T2-weighted FLAIR, and precontrast and postcontrast T1-weighted images. T2-weighted and FLAIR images were acquired using a spin echo sequence with the following parameters: repetition time (TR)/echo time (TE) 3000/100 ms, FOV 240 × 240 mm; matrix, 256 × 256; slice thickness, 4 mm without a gap for T2-weighted image and TR/TE 10000/130 ms, inversion time 2800 ms, FOV 240 × 240 mm; matrix, 256 × 256; and slice thickness, 4 mm without a gap for FLAIR. High-resolution anatomic three-dimensional (3D) volume images were acquired using gradient-echo T1-weighted sequences with the following parameters: TR/TE 9.8/4.6 ms; flip angle, 10°; FOV, 256 × 256 mm; matrix, 512 × 512; and slice thickness, 1 mm with no gap, with and without gadolinium-based contrast agent.

### Image analysis

All images were analyzed on T2 FLAIR imaging and CE-T1WI by two neuroradiologists (H.S.K. and J.E.P.; with 22 and 7 years of clinical experience, respectively, in neuro-oncologic imaging), who were blinded to the clinical information and reference standard.

#### Type of NEL on pre-CCRT examinations

**Fig. 2:**
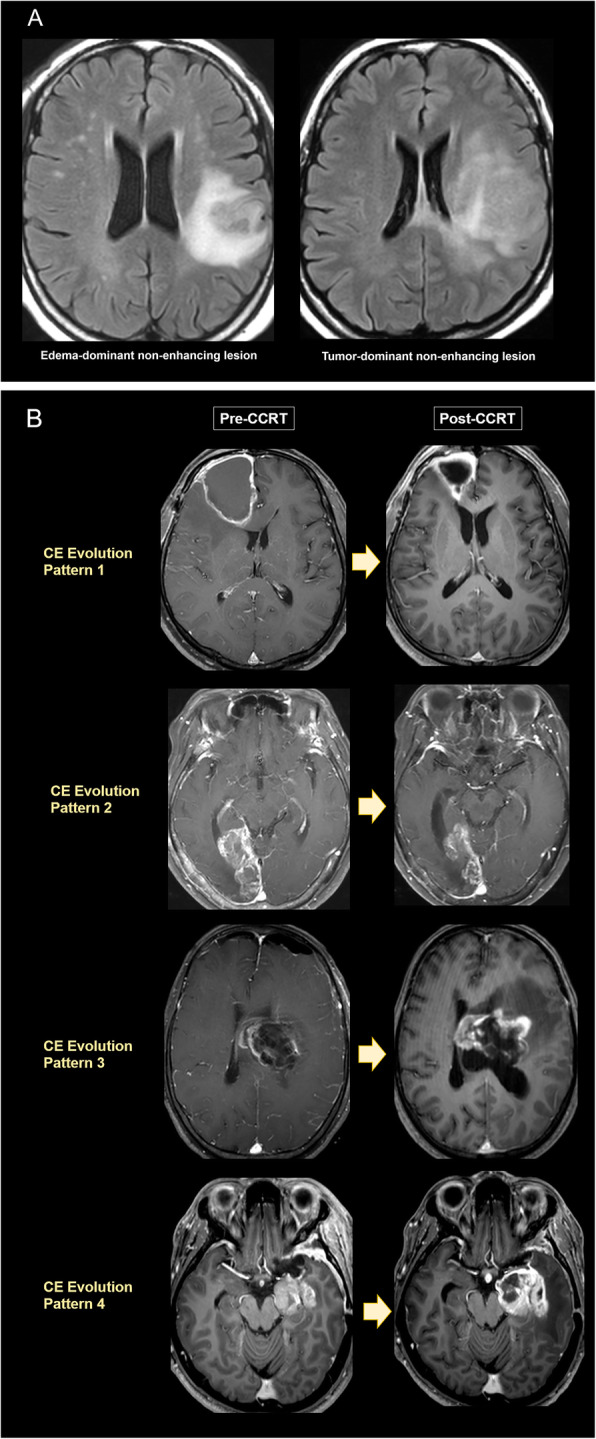
**(A)** Representative patient images for classification of NEL type based on pre-CCRT T2 FLAIR images. Edema-dominant type NEL (left) and tumor-dominant type NEL (right). **(B)** Representative patient images for CE evolution pattern based on pre-CCRT (left) and post-CCRT (right) contrast-enhanced T1-weighted MRI. a) new non-measurable CE after GTR (pattern 1); b) stable or responding residual CE after STR (pattern 2); c) new measurable CE after GTR (pattern 3); and d) enlarging residual CE after STR (pattern 4). NEL = non-enhancing lesion; CE = contrast enhancing lesion; CCRT = concurrent chemoradiotherapy; GTR = gross total resection; STR = subtotal resection

NELs, visualized as T2 FLAIR hyperintense lesions on pre-CCRT MRI, were classified as edema-dominant or tumor-dominant type. Characteristic MRI features for tumor-dominant type NEL were relatively mild FLAIR hyperintensity, gray matter involvement, eccentric extension not accounted for by anatomic constraints, focal parenchymal expansion, and mass effect [14–16] (Fig. 2A). The two neuroradiologists evaluated MRI results independently, and agreement was calculated. For multivariate analysis, disagreements were reconciled by consensus.

#### Evolution pattern of CE from pre- to post-CCRT examinations

CEs, visualized on CE-T1WI, were categorized by two readers in consensus as (i) new non-measurable CE after gross total resection (GTR) (pattern 1); (ii) stable or responding residual CE after subtotal resection (STR) (pattern 2); (iii) new measurable CE after GTR (pattern 3); and (iv) and enlarging residual CE after STR (pattern 4) (Fig. 2B). Measurable CE was defined as bidimensional CE with clearly defined margins, with two perpendicular diameters of at least 10 mm, visible on two or more axial slices [4]. Non-measurable CE was defined as the absence of clearly defined margins, unidimensional measurable lesions, or lesion maximal perpendicular diameters < 10 mm [4]. Enlarging CE was defined as a > 25% increase from baseline in the sum of the products of perpendicular diameters of CE.

### Statistical analysis

The demographic and clinical characteristics of the edema-dominant and tumor-dominant NEL groups were assessed by Student’s t-test or the chi-square test, as appropriate.

#### Inter-reader agreement

The inter-reader agreement on NEL type was assessed by using kappa statistics.

### Prediction of TTP

The effects of NEL type and CE evolution pattern on TTP and OS were evaluated by the Kaplan–Meier method and compared by log-rank tests and by Cox proportional hazard regression models. The effects of CE evolution pattern on TTP and OS in the two NEL subgroups were also analyzed by the Kaplan–Meier method and compared by log-rank tests.

#### Prediction of pseudoprogression

Differences in pseudoprogression rates according to NEL type and CE evolution pattern were assessed by chi-square tests and multivariable logistic regression analysis, after adjusting for age, sex, and Karnofsky performance status (KPS) at baseline. The diagnostic accuracy of predicting pseudoprogression was analyzed according to NEL type and CE evolution pattern, both independently and as a combination of NEL type and CE evolution pattern.

All statistical analyses were performed using MedCalc version 19.2.1 and R version 3.6.3 statistical software, with *P*-values <.05 considered statistically significant.

## Results

### Patient demographics

Of 283 potentially eligible patients, 43 patients with IDH-mutant type or unknown IDH status, 65 patients without appropriate pre-CCRT or sequential follow-up MRI, and 89 patients with regressed CE on post-CCRT MRI were excluded. Finally, 86 patients, 49 (57.0%) men and 37 (43.0%) women, of mean age 58 years, were included in the analysis. Of these 86 patients, 41 (47.7%) were classified as edema-dominant type NEL group and 45 (52.3%) as tumor-dominant type NEL group. Final diagnosis for pseudoprogression and true progression was based on pathologic confirmation for 16 (18.6%) patients and clinicoradiologic follow-up for 70 (81.4%) patients. There were no significant differences between groups of patients with edema-dominant type and tumor-dominant type NEL in age at diagnosis, sex, KPS at baseline, and O6-methylguanine-DNA methyltransferase (MGMT) promoter methylation status (Table 1). On the other hand, edema-dominant NEL had a higher rate of GTR (*P* = .02) than tumor-dominant NEL group.

**Table 1:** Demographic Characteristics of the Study Population

	Edema-dominant NEL Group (***n*** = 41)	Tumor-dominant NEL Group (***n*** = 45)	***P***
Age, years, median (range)	59 (31–81)	57 (31–77)	.362
No. of women patients (% of patients)	15 (36.6%)	22 (48.9%)	.253
KPS at baseline ≥70 (% of patients)	38 (92.7%)	40 (88.9%)	.548
MGMT promoter methylation (positive/negative/missing)	18/19/4	16/19/10	.285
Type of surgery (biopsy/STR/GTR)	0/4/37	3/12/30	**.023**
No. with pseudoprogression (% of patients)	21 (51.2%)	13 (28.9%)	**.036**
No. with true progression (% of patients)	20 (48.8%)	32 (71.1%)	.119
Median TTP, days	257 (95% CI: 205.9–305.5)	105 (95% CI: 98.5–153.6)	**.042**

*NEL* non-enhancing lesion, *IDH* isocitrate dehydrogenase, *KPS* Karnofsky performance status, *MGMT* O6-methylguanine-DNA methyltransferase, *STR* subtotal resection, *GTR* gross total resection, *CI* confidence interval, *TTP* time to progression

### Prediction of Pseudoprogression

Of the 86 patients, 34 (39.5%) showed pseudoprogression. In patients with CE evolution pattern 3 and 4, pseudoprogression rate was lower in the tumor-dominant type than in the edema-dominant type NEL group (chi-square = 3.940, *P* = .047). Multivariate analyses that included age, sex, KPS, NEL type, and CE evolution pattern showed that NEL type was the only independent predictive marker for pseudoprogression (odds ratio [OR] = 0.26, 95% CI = 0.00–0.52, *P* = .046). CE evolution pattern was not associated with pseudoprogression (*P* = .407). Accuracy of a combination of NEL type and CE evolution patterns in predicting pseudoprogression was shown in the Supplementary Table 1. The edema-dominant type NEL could predict pseudoprogression with an accuracy of 62.8%, while the tumor-dominant type NEL could do the same with an accuracy of only 38.4%. After combining NEL type and CE evolution pattern, the accuracy of predicting pseudoprogression was highest in edema-dominant type NEL with CE evolution pattern 3 (62.8%), followed by edema-dominant type NEL with CE evolution pattern 1 (61.6%), tumor-dominant type NEL with CE evolution pattern 1 (60.5%), edema-dominant type NEL with CE evolution pattern 2 (59.3%), edema-dominant type NEL with CE evolution pattern 4 (59.3%), tumor-dominant type NEL with CE evolution pattern 2 (59.3%), tumor-dominant type NEL with CE evolution pattern 4 (53.5%), and tumor-dominant type NEL with CE evolution pattern 3 (46.5%).

### Independent prediction of TTP and OS based on NEL type and CE evolution pattern

The kappa value for interobserver agreement on type of NEL was 0.81 (95% confidence interval [CI] = 0.69–0.94), indicating almost perfect agreement.

Table 2 summarizes the association between imaging predictors and TTP. Evaluation of all study patients showed that TTP was significantly shorter in patients with tumor-dominant type than edema-dominant type NEL (hazard ratio [HR] = 2.03, *P* = .005). OS tended to be shorter in patients with tumor-dominant type than with edema-dominant type NEL. However, this difference did not reach statistical significance (HR = 1.37, *P* = .196) (Supplementary Fig. 1).

**Table 2:** Univariate Cox Proportional Hazard Analysis of Prediction of TTP in Patients Stratified by NEL Type and CE Evolution Pattern

Entire Patients (***n*** = 86)	Hazard Ratio	95% CI	***P***
**NEL type**			
Tumor-dominant	2.03	1.24–3.32	**.005**
Edema-dominant	1		
**CE evolution pattern**			
Enlarging residual CE (pattern 4)	4.33	2.02–9.28	**<.001**
New measurable CE (pattern 3)	3.00	1.63–5.54	**<.001**
Stable or responding residual CE (pattern 2)	2.44	0.80–7.28	.117
New non-measurable CE (pattern 1)	1		
**Patients with edema-dominant NEL (*n =*** **41)**			
Enlarging residual CE (pattern 4)	6.92	1.71–27.93	**.007**
New measurable CE (pattern 3)	5.36	2.10–13.66	**<.001**
Stable or responding residual CE (pattern 2)	2.63	0.31–21.98	.372
New non-measurable CE (pattern 1)	1		

*TTP* time to progression, *NEL* non-enhancing lesion, *CE* contrast enhancement, *CI* confidence interval

Stratification by CE evolution pattern showed that TTP was shortest in patients with enlarging residual CE after STR (pattern 4; HR = 4.33, *P* < .001), followed by new measurable CE after GTR (pattern 3; HR = 3.00, *P* < .001), stable or responding residual CE after STR (pattern 2; HR = 2.44, *P* = .117), and new non-measurable CE after GTR (pattern 1; *P* < .001). Stratification by CE evolution pattern revealed that the shortest OS was found in patients with pattern 2 (HR = 3.71, *P* = .009), followed by pattern 4 (HR = 2.74, *P* = .04), pattern 3 (HR = 2.50, *P* = .005), and pattern 1 (*P* < .001) (Supplementary Fig. 2).

### Prediction of TTP and OS based on the relationship between NEL type and CE evolution pattern

#### Relationship between NEL type and CE evolution pattern

**Fig. 3:**
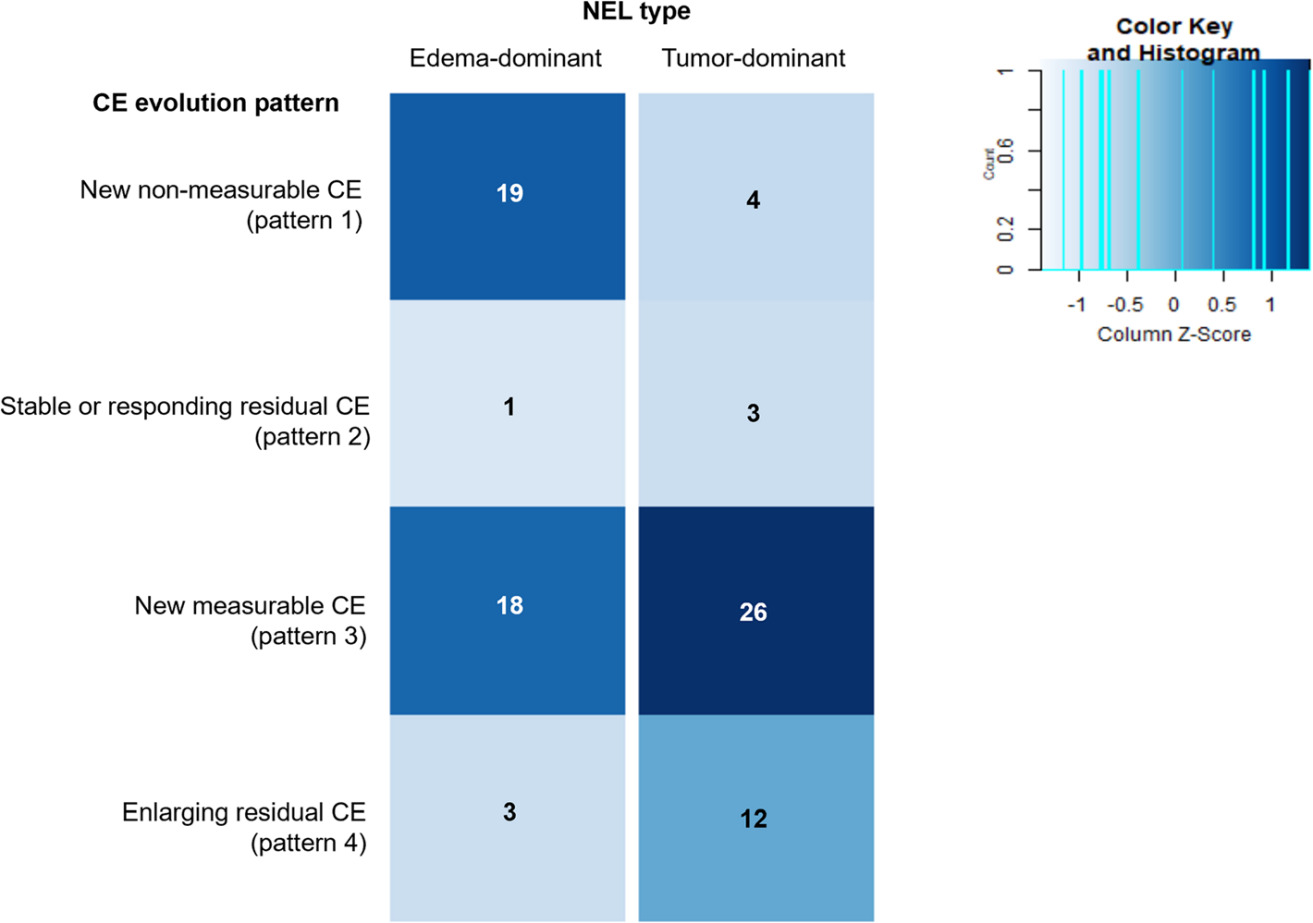
Heatmap of CE evolution pattern in patients with edema-dominant type and tumor-dominant type NEL. CE = contrast enhancement; NEL = non-enhancing lesion

Of the 41 patients with edema-dominant type NEL, 19 were classified as new non-measurable CE after GTR (pattern 1), one as stable or responding residual CE after STR (pattern 2), 18 as new measurable CE after GTR (pattern 3), and three as enlarging residual CE after STR (pattern 4). Of the 45 patients with tumor-dominant type NEL, four, three, 26, and 12 were classified as having 1, 2, 3, and 4 CE evolution pattern, respectively. NEL type was significantly associated with CE evolution pattern (chi-square = 11.963, *P* = .008, Fig. 3). A high proportion of patients with edema-dominant type NEL had CE evolution pattern 1 and 3, whereas a proportion of patients with tumor-dominant type NEL had CE evolution pattern 3 and 4.

#### Prediction of TTP and OS based on NEL type and CE evolution pattern

Subgroup analysis showed that TTP differed significantly in patients with edema-dominant type NEL according to CE evolution pattern. TTP was shortest in patients with CE evolution pattern 4 (HR = 6.92, *P* = .007), followed by pattern 3 (HR = 5.36, *P* < .001), pattern 2 (HR = 2.63, *P* = .372), and pattern 1 (log-rank, *P* = .001). TTP in patients with tumor-dominant type NEL, however, did not differ significantly according to CE evolution pattern (log-rank, *P* = .528).

**Fig. 4:**
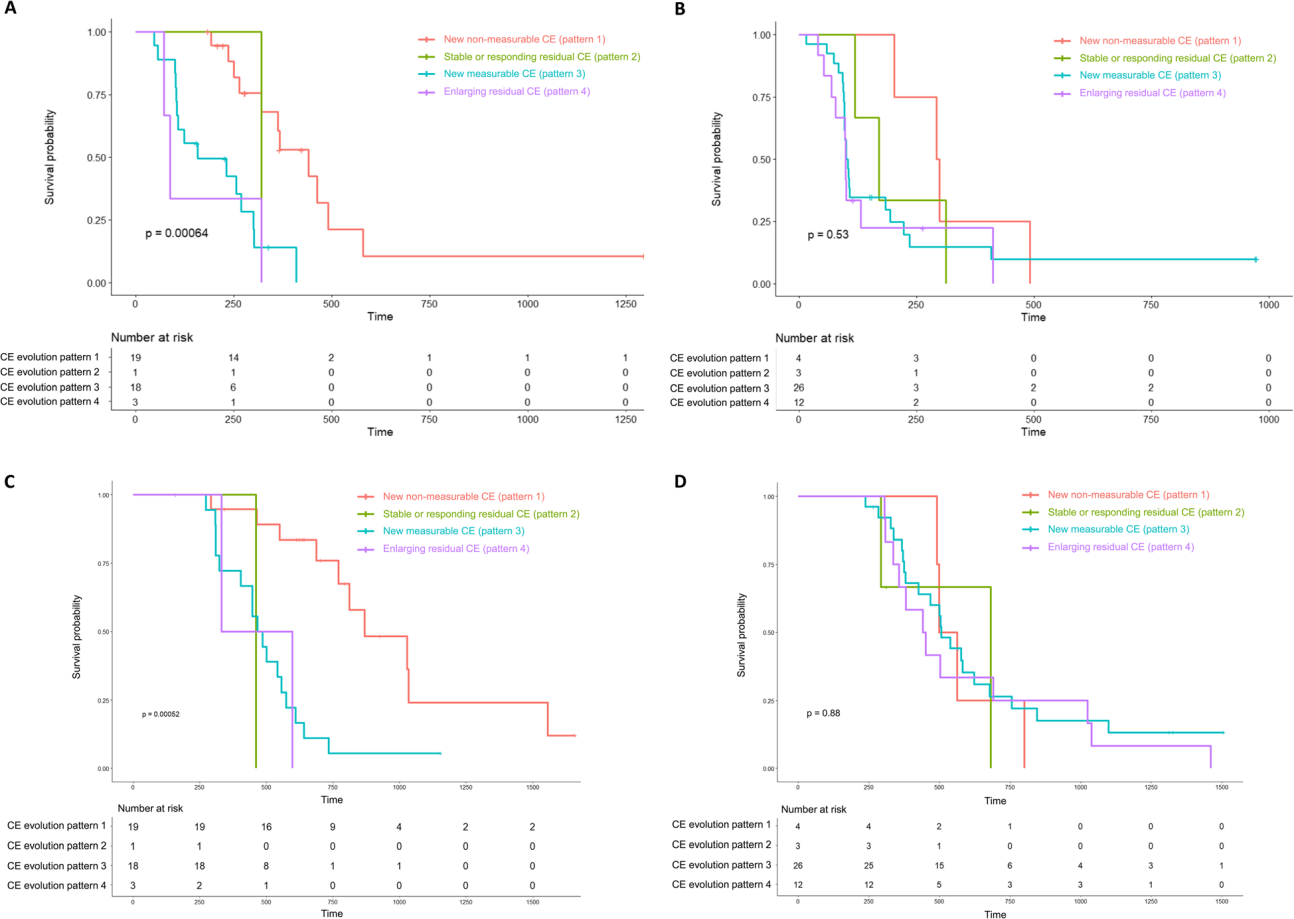
Prediction of TTP (A, B) and OS (C, D) based on NEL type and CE evolution pattern in patients with IDH wild-type glioblastoma. **(A)** Kaplan–Meier analysis of TTP in patients with edema-dominant type NEL stratified by CE evolution pattern (log-rank, *P* = .001). **(B)** Kaplan–Meier analysis of TTP in patients with tumor-dominant type NEL stratified by CE evolution pattern (log-rank, *P* = .528). **(C)** Kaplan–Meier analysis of OS in patients with edema-dominant type NEL stratified by CE evolution pattern (log-rank, *P* = .001). **(D)** Kaplan–Meier analysis of OS in patients with tumor-dominant type NEL stratified by CE evolution pattern (log-rank, *P* = .879). TTP = time to progression; OS = overall survival; NEL = non-enhancing lesion; CE = contrast enhancement; IDH = isocitrate dehydrogenase

With OS, the same trend was observed. In patients with edema-dominant type NEL, OS was stratified by CE evolution pattern, shortest in patients with pattern 2 (HR = 9.11, *P* = .04), followed by pattern 4 (HR = 6.2, *P* = .023), pattern 3 (HR = 4.65, *P* < .001), and pattern 1 (log-rank, *P* < .001). OS with tumor-dominant type NEL did not differ in accordance with CE evolution pattern (log-rank, *P* = .879) (Fig. 4).

## Discussion

This study analyzed the ability of type of peritumoral NEL and evolution pattern of CE in response to CCRT to predict pseudoprogression, TTP, and OS in patients with IDH wild-type glioblastoma. TTP was found to be shorter and pseudoprogression rate lower in patients with tumor-dominant than edema-dominant type NEL. Of the four evolution patterns of CE, enlarging residual CE after STR (pattern 4) showed shortest TTP while stable residual CE after STR (pattern 2) showed shortest OS. Interestingly, both TTP and OS was better predicted by CE evolution pattern and NEL type than by either alone. TTP and OS could be stratified by CE evolution pattern in patients with edema-dominant, but not tumor-dominant, NEL. Taken together, these results suggested that, when combined with CE evolution and extent of surgical resection, NEL type improves the prediction of pseudoprogression and helps to further stratify TTP and OS.

Several recent studies have suggested that NEL may be a prognostic marker in patients with glioblastoma [9, 16]. For example, a multicenter study found that high postoperative residual NEL volume was significantly associated with poor overall survival [9]. Histopathologic examination showed that the content of viable tumor cells was higher in non-contrast enhancing tumors than in contrast enhancing tumors or necrotic tumor components [6]. Nonetheless, association of residual NEL (subdivided into edema- and tumor-dominant types) with treatment remains poorly understood. In our study, patients with tumor-dominant NEL type on pre-CCRT examination had higher rates of true progression at the appearance of contrast enhancing lesions, as well as shorter TTP. This NEL type was a stronger predictor of pseudoprogression than CE evolution pattern during CCRT. In the clinical setting, the more aggressive management of NEL, with neurosurgical resection or radiosurgery, has shown greater prognostic benefit than management of the contrast enhancing lesion alone [23, 24]. However, complete resection of NEL is difficult and could increase the risk of postoperative neurological deficits [25]. This study showed the importance of distinguishing tumor-dominant type from edema-dominant type NEL and the selective aggressive management of tumor-dominant type may prolong survival. In addition, morphologic categorization of NEL type showed almost perfect interobserver agreement, suggesting that NEL type can become a useful and reproducible biomarker on anatomical MRI.

We categorized CE evolution pattern in response to CCRT based on surgical extent, change in lesion size, and lesion measurability, all of which are important variables in predicting tumor response and prognosis [7, 18, 26]. Although extent of residual CE has been associated with survival [17, 18], few studies have evaluated both residual CE and CE evolution pattern following CCRT. Our study showed that prognosis was worst in patients with residual CE compared to new CE, in agreement with studies showing the importance of the extent of residual CE [17, 18]. Combining surgical extent with CE evolution may therefore improve patient prognosis. Our study revealed that the TTP was shortest in patients with enlarging residual CE, while OS was shortest in patients with stable or responding residual CE. We speculated that although enlarging residual CE could be a direct marker for early tumor progression, pseudoprogression might account for a significant portion of it and affect the long-term outcomes of OS. Tumor heterogeneity of glioblastoma is an important reason for treatment failure, and tumor cells that survive initial therapy mainly cause tumor re-growth or recurrence [27]. The remaining tumor cells in stable or responding CE might lead to treatment resistance and ultimately be responsible for poor patient prognosis.

CE evolution pattern were significantly associated with prognosis, TTP, and OS in patients with edema-dominant, but not tumor-dominant NEL [27]. To our knowledge, no previous studies have investigated the relationship between NEL type and CE evolution pattern, nor assessed the ability of both together to predict prognosis. Our study showed a significant association between NEL type and CE evolution pattern. CE evolution pattern 3 and 4 were observed more frequently in tumor-dominant than edema-dominant type NEL, suggesting that NEL type should be considered when determining CE evolution pattern, and that both should be considered in predicting patient prognosis.

This study had several limitations in addition to those due to its retrospective nature. First, histological confirmation was not possible at the time of radiographic progression because of the invasiveness of these tumors. Second, the number of patients with IDH wild-type glioblastoma was relatively small. Third, this study did not include the results of advanced MRI, including DWI and perfusion-weighted imaging, which increase the diagnostic accuracy of pseudoprogression [28–31]. However, lack of standardization of advanced imaging protocols has prevented their use as imaging biomarkers in multicenter practices. Our study only included the results of conventional MRI, which is generally accepted in multicenter practices. Fourth, volumetric measurement of CE had not been applied. Further studies are warranted before applying our results to daily clinical practice.

## Conclusion

In conclusion, the addition of peritumoral NEL type to CE evolution pattern improves prediction of pseudoprogression and helps to further stratify TTP and OS in patients with IDH wild-type glioblastoma. Edema-dominant NEL showed a greater association with CE-based survival stratification and pseudoprogression, whereas tumor-dominant NEL was an independent predictor of shorter TTP. This determination of NEL type, together with CE evolution pattern, may further stratify TTP and OS and may become a helpful imaging biomarker for refining RANO criteria.

## Supplementary Information

**Additional file 1: Supplementary Table 1.** Accuracy of a combination of non-enhancing lesions (NEL) type and contrast enhancement (CE) evolution patterns in predicting pseudoprogression. **Supplementary Fig. 1. (A)** Kaplan–Meier analysis of time to progression (TTP) in patients with tumor-dominant and edema-dominant types of non-enhancing lesions (NEL) inIDH wild-type glioblastoma. The TTP was significantly shorter in patients with tumor-dominant than edema-dominant NEL (hazard ratio [HR] = 2.03; log-rank, *P* = .005).). **(B)** Kaplan–Meier analysis of overall survival (OS) in patients with tumor-dominant and edema-dominant types of NEL in IDH wild-type glioblastoma. The OS tends to be shorter in patients with tumor-dominant than in patients with edema-dominant NEL, but the difference did not reach statistical significance (HR = 1.37, *P* = .196). **Supplementary Fig. 2. (A)** Kaplan–Meier analysis of time to progression (TTP) based on contrast enhancement (CE) evolution patterns in patients with IDH wild-type glioblastoma. TTP was shortest in patients with enlarging residual CE after STR (subtotal resection) (pattern 4; HR = 4.33, *P* < .001), followed by new measurable CE after GTR (gross total resection) (pattern 3; HR = 3.00, *P* < .001), stable or responding residual CE after STR (pattern 2; HR = 2.44, *P* = .117), and new non-measurable CE after GTR (pattern 1; *P* < .001). **(B)** Kaplan–Meier analysis of overall survival (OS) based on CE evolution patterns in patients with IDH wild-type glioblastoma. OS was shortest in patients with pattern 2 (HR = 3.71, *P* = .009), followed by pattern 4 (HR = 2.74, *P* = .04), pattern 3 (HR = 2.50, *P* = .005), and pattern 1 (*P* < .001).

## Data Availability

The datasets generated and/or analysed during the current study are not publicly available due to the hospital’s policy but are available from the corresponding author on reasonable request.

## Abbreviations

IDH: Isocitrate dehydrogenase
CCRT: Concurrent chemoradiotherapy
CE: Contrast enhancement
NEL: Non-enhancing lesion
SD: Stable disease
PD: Progressive disease
TTP: Time to progression
OS: Overall survival
