# Supplementary material for: Refinement of response assessment in neuro-oncology (RANO) using non-enhancing lesion type and contrast enhancement evolution pattern in IDH wild-type glioblastomas
Source: BMC Cancer. 2021 Jun 1;21:654. doi: 10.1186/s12885-021-08414-2 (PMC8170938; doi:10.1186/s12885-021-08414-2)
Supplement: Supplementary file 1 — Additional file 1: Supplementary Table 1. Accuracy of a combination of non-enhancing lesions (NEL) type and contrast enhancement (CE) evolution patterns in predicting pseudoprogression. Supplementary Fig. 1. (A) Kaplan–Meier analysis of time to progression (TTP) in patients with tumor-dominant and edema-dominant types of non-enhancing lesions (NEL) inIDH wild-type glioblastoma. The TTP was significantly shorter in patients with tumor-dominant than edema-dominant NEL (hazard ratio [HR] = 2.03; log-rank, P = .005).). (B) Kaplan–Meier analysis of overall survival (OS) in patients with tumor-dominant and edema-dominant types of NEL in IDH wild-type glioblastoma. The OS tends to be shorter in patients with tumor-dominant than in patients with edema-dominant NEL, but the difference did not reach statistical significance (HR = 1.37, P = .196). Supplementary Fig. 2. (A) Kaplan–Meier analysis of time to progression (TTP) based on contrast enhancement (CE) evolution patterns in patients with IDH wild-type glioblastoma. TTP was shortest in patients with enlarging residual CE after STR (subtotal resection) (pattern 4; HR = 4.33, P < .001), followed by new measurable CE after GTR (gross total resection) (pattern 3; HR = 3.00, P < .001), stable or responding residual CE after STR (pattern 2; HR = 2.44, P = .117), and new non-measurable CE after GTR (pattern 1; P < .001). (B) Kaplan–Meier analysis of overall survival (OS) based on CE evolution patterns in patients with IDH wild-type glioblastoma. OS was shortest in patients with pattern 2 (HR = 3.71, P = .009), followed by pattern 4 (HR = 2.74, P = .04), pattern 3 (HR = 2.50, P = .005), and pattern 1 (P < .001). [file 12885_2021_8414_MOESM1_ESM.docx]

**Supplementary Materials for**

**Refinement of Response Assessment in Neuro-Oncology (RANO) using Non-Enhancing Lesion Type and Contrast Enhancement Evolution Pattern in IDH Wild-type Glioblastomas**

Hye Hyeon Moon, M.D., Ho Sung Kim, M.D., Ph.D., Ji Eun Park, M.D., Ph.D., Young-Hoon Kim, M.D., Ph.D., Jeong Hoon Kim, M.D., Ph.D.

From the Department of Radiology and Research Institute of Radiology (H.H.M., H.S.K., J.E.P.) and Department of Neurosurgery (J.H.K., Y.H.K.), University of Ulsan College of Medicine, Asan Medical Center, 86 Asanbyeongwon-Gil, Songpa-Gu, Seoul 05505, Republic of Korea

Corresponding author: Ho Sung Kim, M.D., Ph.D.

Department of Radiology and Research Institute of Radiology, University of Ulsan College of Medicine, Asan Medical Center, 43 Olympic-ro 88, Songpa-Gu, Seoul 05505, Korea

Phone: 82-2-3010-5682

E-mail address: radhskim@gmail.com

**Supplementary Table 1:** Accuracy of a combination of non-enhancing lesions (NEL) type and contrast enhancement (CE) evolution patterns in predicting pseudoprogression.

| NEL type | CE evolution pattern | No. of Patients | Accuracy (%) |
| --- | --- | --- | --- |
| Edema-dominant | CE evolution pattern 1 | 19 | 61.6 |
|  | CE evolution pattern 2 | 1 | 59.3 |
|  | CE evolution pattern 3 | 18 | 62.8 |
|  | CE evolution pattern 4 | 3 | 59.3 |
| Tumor-dominant | CE evolution pattern 1 | 4 | 60.5 |
|  | CE evolution pattern 2 | 3 | 59.3 |
|  | CE evolution pattern 3 | 26 | 46.5 |
|  | CE evolution pattern 4 | 12 | 53.5 |

**Supplementary Figure 1: (A)** Kaplan–Meier analysis of time to progression (TTP) in patients with tumor-dominant and edema-dominant types of non-enhancing lesions (NEL) inIDH wild-type glioblastoma. The TTP was significantly shorter in patients with tumor-dominant than edema-dominant NEL (hazard ratio [HR] = 2.03; log-rank, *P* = .005). ). **(B)** Kaplan–Meier analysis of overall survival (OS) in patients with tumor-dominant and edema-dominant types of NEL in IDH wild-type glioblastoma. The OS tends to be shorter in patients with tumor-dominant than in patients with edema-dominant NEL, but the difference did not reach statistical significance (HR = 1.37, P = .196).

**(A)**


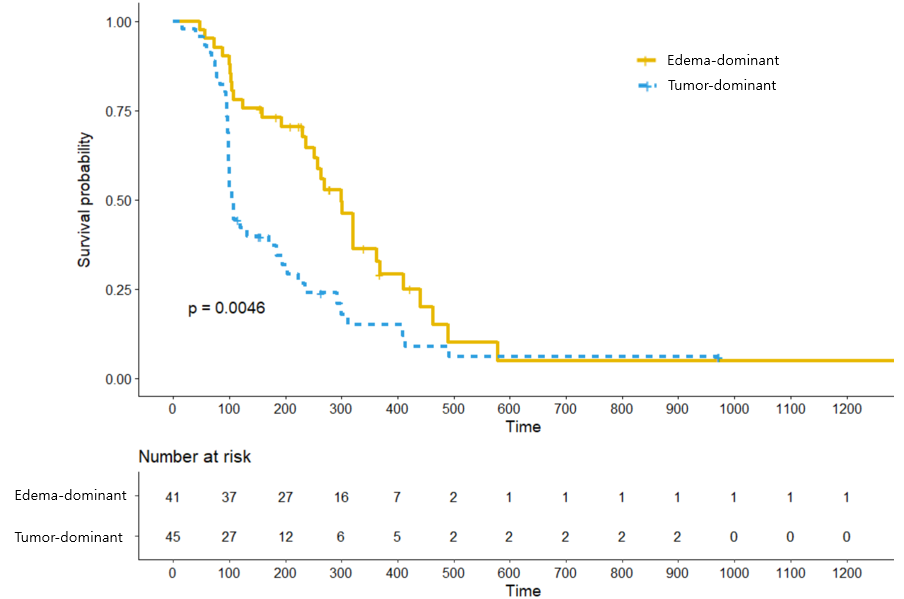


**(B)**

**
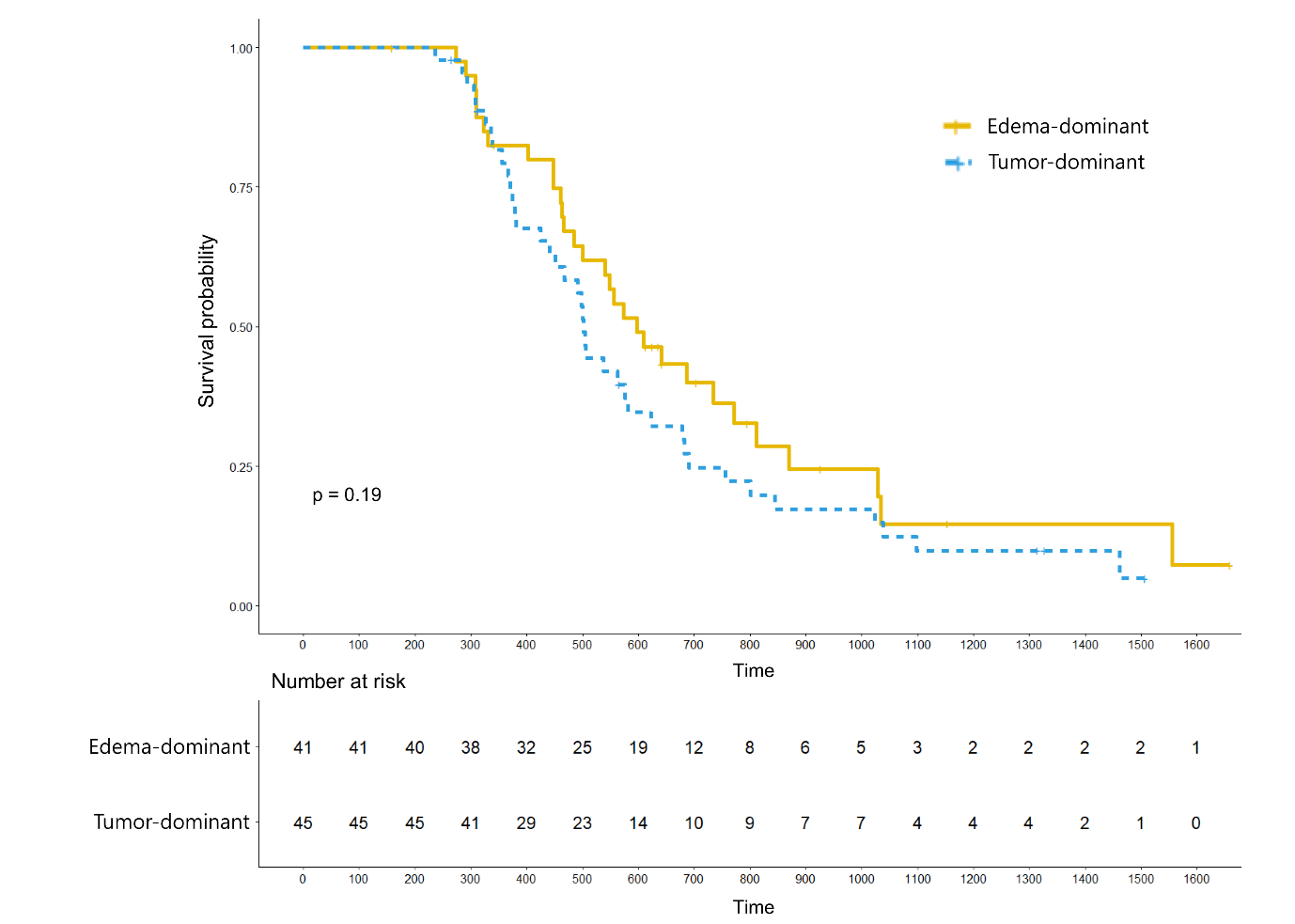
**

**Supplementary Figure 2: (A)** Kaplan–Meier analysis of time to progression (TTP) based on contrast enhancement (CE) evolution patterns in patients with IDH wild-type glioblastoma. TTP was shortest in patients with enlarging residual CE after STR (subtotal resection) (pattern 4; HR = 4.33, *P* < .001), followed by new measurable CE after GTR (gross total resection) (pattern 3; HR = 3.00, *P* < .001), stable or responding residual CE after STR (pattern 2; HR = 2.44, *P* = .117), and new non-measurable CE after GTR (pattern 1; *P* < .001). **(B)** Kaplan–Meier analysis of overall survival (OS) based on CE evolution patterns in patients with IDH wild-type glioblastoma. OS was shortest in patients with pattern 2 (HR = 3.71, P = .009), followed by pattern 4 (HR = 2.74, P = .04), pattern 3 (HR = 2.50, P = .005), and pattern 1 (P < .001).

**(A)**
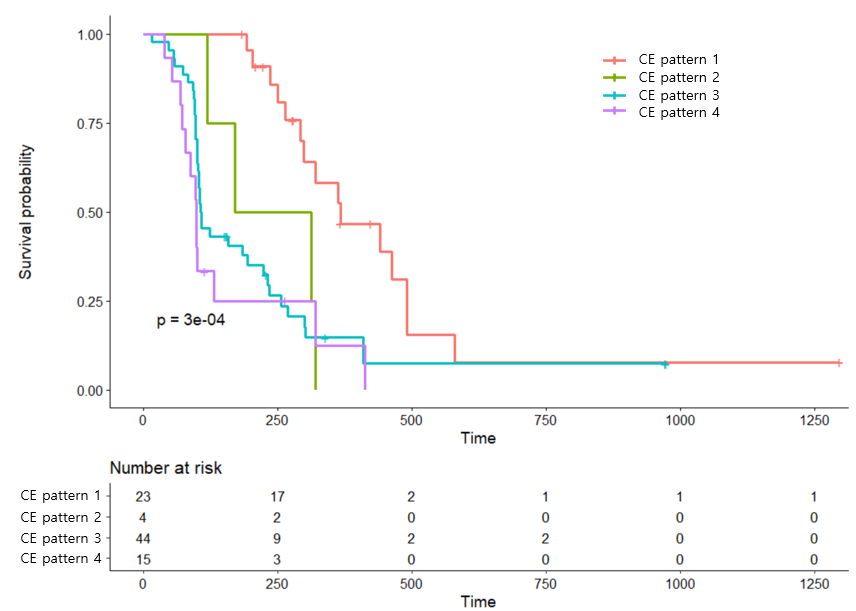


**(B)**

**
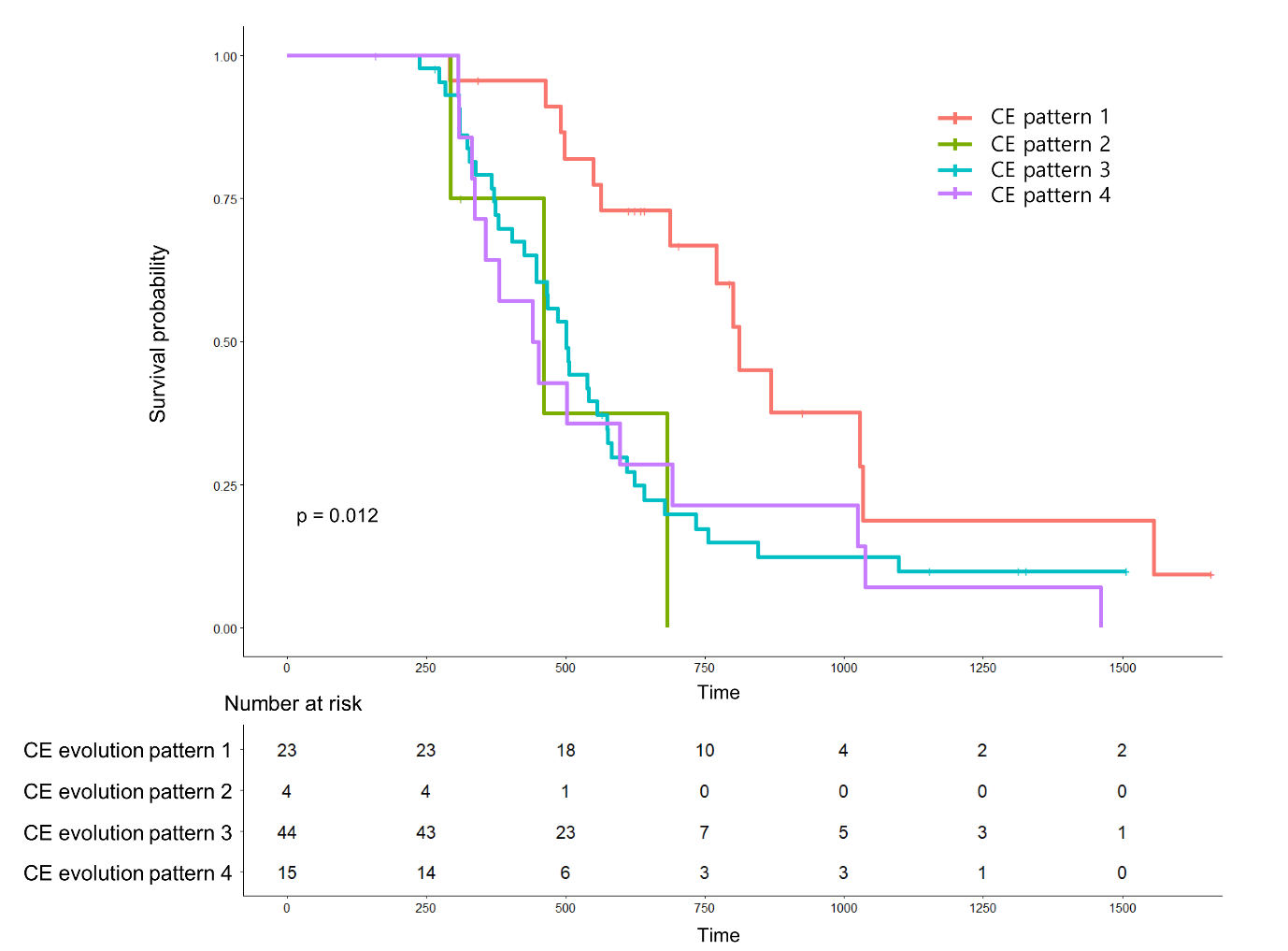
**
